# Publisher Correction: Quantized topological pumping of solitons in nonlinear photonics and ultracold atomic mixtures

**DOI:** 10.1038/s41467-022-34143-6

**Published:** 2022-11-17

**Authors:** Nader Mostaan, Fabian Grusdt, Nathan Goldman

**Affiliations:** 1 Department of Physics and Arnold Sommerfeld Center for Theoretical Physics (ASC), Ludwig-Maximilians-Universität München, Theresienstr. 37, D-80333 München, Germany; 2 Munich Center for Quantum Science and Technology (MCQST), Schellingstr. 4, D-80799 München, Germany; 3 CENOLI, Université Libre de Bruxelles, CP 231, Campus Plaine, B-1050 Brussels, Belgium

**Keywords:** Ultracold gases, Solitons, Topological matter, Bose-Einstein condensates, Solitons

Correction to: *Nature Communications* 10.1038/s41467-022-33478-4, published online 11 October 2022

The original version of this Article contained an error in the first two sentences of the Acknowledgements section. The section only included initials of the people acknowledged “We are glad to thank B.O., A.B., N.E., S.-P.G., F.L., and S.M. for useful discussions. We also acknowledge M.J. and M.C.R. for fruitful discussions, for sharing their results in the course of our numerical studies and for enlightening us on the negligible role of band mixing.”

The correct version includes the full last names “We are glad to thank B. Oblak, A. Bedroya, N. Englebert, S.-P. Gorza, F. Leo and S. Mukherjee for useful discussions. We also acknowledge M. Jürgensen and M. C. Rechtsman for fruitful discussions, for sharing their results in the course of our numerical studies and for enlightening us on the negligible role of band mixing.”

This has been corrected in both the PDF and HTML versions of the Article.

